# Region-Specific Sensitivity of Anemophilous Pollen Deposition to Temperature and Precipitation

**DOI:** 10.1371/journal.pone.0104774

**Published:** 2014-08-18

**Authors:** Timme H. Donders, Kimberley Hagemans, Stefan C. Dekker, Letty A. de Weger, Pim de Klerk, Friederike Wagner-Cremer

**Affiliations:** 1 Palaeoecology, Department of Physical Geography, Faculty of Geosciences, Utrecht University, Laboratory of Palaeobotany and Palynology, Utrecht, The Netherlands; 2 Department of Environmental Sciences, Copernicus Institute, Faculty of Geosciences, Utrecht University, Utrecht, The Netherlands; 3 Department of Pulmonology, Leiden University Medical Centre, Leiden, The Netherlands; 4 Botany section, Staatliches Museum für Naturkunde Karlsruhe, Karlsruhe, Germany; University of Colorado, United States of America

## Abstract

Understanding relations between climate and pollen production is important for several societal and ecological challenges, importantly pollen forecasting for pollinosis treatment, forensic studies, global change biology, and high-resolution palaeoecological studies of past vegetation and climate fluctuations. For these purposes, we investigate the role of climate variables on annual-scale variations in pollen influx, test the regional consistency of observed patterns, and evaluate the potential to reconstruct high-frequency signals from sediment archives. A 43-year pollen-trap record from the Netherlands is used to investigate relations between annual pollen influx, climate variables (monthly and seasonal temperature and precipitation values), and the North Atlantic Oscillation climate index. Spearman rank correlation analysis shows that specifically in *Alnus*, *Betula*, *Corylus*, *Fraxinus*, *Quercus* and *Plantago* both temperature in the year prior to (T_-1_), as well as in the growing season (T), are highly significant factors (T_April_ r_s_ between 0.30 [P<0.05[ and 0.58 [P<0.0001]; T_Juli-1_ rs between 0.32 [P<0.05[ and 0.56 [P<0.0001]) in the annual pollen influx of wind-pollinated plants. Total annual pollen prediction models based on multiple climate variables yield R^2^ between 0.38 and 0.62 (P<0.0001). The effect of precipitation is minimal. A second trapping station in the SE Netherlands, shows consistent trends and annual variability, suggesting the climate factors are regionally relevant. Summer temperature is thought to influence the formation of reproductive structures, while temperature during the flowering season influences pollen release. This study provides a first predictive model for seasonal pollen forecasting, and also aides forensic studies. Furthermore, variations in pollen accumulation rates from a sub-fossil peat deposit are comparable with the pollen trap data. This suggests that high frequency variability pollen records from natural archives reflect annual past climate variability, and can be used in palaeoecological and -climatological studies to bridge between population- and species-scale responses to climate forcing.

## Introduction

Pollen production by wind pollinated (anemophilous) plants is characterized by inter-annual variation [1]. This variation is not random but related to biological processes (e.g. mast cycles, [2] and changes in vegetation dynamics such as tree line fluctuations [3]. Part of the variation is caused by climatic conditions, particularly the character of the seasonal cycle [3], [4] but also lower-frequency climate variability [5]. Identification of the strength, sign and type of effect of climatic variables on the annual pollen deposition is significant in multiple fields of research, ranging from palaeoecology and palaeoclimate [6], global change biology [7], plant ecology [8], to allergology [9], and forensics [10].

Firstly, if annual pollen production is influenced by climate variables, then high resolution records of fossil pollen have the potential for quantitative reconstruction of past climate conditions on annual to decadal time scales [6], [11]–[13]. Traditionally, palynological studies focus on population-scale successional changes to study variations in past vegetation cover and associated climate change [14]. A higher resolution is needed for understanding past variability of climate systems that vary on annual to decadal timescales such as the North Atlantic Oscillation (NAO) for Europe and North America [15], and climatic oscillations in other parts of the world. Beside vegetation succession, annual-scale studies of pollen production and deposition rates allow us to assess the influence of climatic factors on such short timescales, and identify how such signals are preserved in natural archives that are the source for vegetation and derived climate reconstructions.

Secondly, in allergology accurate and timely prediction of seasonal pollen production is important for sufferers of pollinosis (hay fever) [9]. Tree pollen allergens affect health in up to 15% of the population [2].While much effort is directed at observed and future changes on the timing of pollen release e.g. [16], [17], or masting effects [2], it is vital to also establish the exact relation between total annual pollen production and climate variables to improve predictions of seasonal pollen concentrations [18]. Global climate changes will likely affect the intensity of the pollen season e.g. [7], [19] whereby region-specific studies of climate parameters relevant for pollen production and deposition will lead to better long-term pollinosis scenarios. This involves quantification of both the annual-scale variability and decadal-scale climatic trends and pollen production to improve the long-term pollen predictions and region-specific seasonal forecast of pollinosis.

Thirdly, forensic studies benefit from well-documented relations between pollen deposition and climate parameters to reduce uncertainty between concentration and composition of pollen deposition and pollen assemblages collected from crime scenes [10]. As for pollen-based climate reconstructions, forensic palynological studies need to take into account both annual climatic conditions, as well as differential preservation (taphonomy) of pollen in soils compared to the atmospheric composition as factors that influence sample comparison.

In this study we aim to improve our understanding of the region-specific response of annual pollen deposition to climate variables for common anemophilous pollen types in NW Europe. We assess the regional variability between pollen trap records, the strength and type of climate-pollen correlations, the robustness of the correlations for the purpose of seasonal forecasts and climate reconstructions, and the preservation of the annual variability in natural peat archives in comparison to the trap data.

While timing of pollen release is commonly known to be climate dependent [17], [20]–[22], the annual pollen production is also affected in several ways [3]–[5], [23], [24]. In most trees, reproductive structures are formed during the summer in the year prior to flowering [1], [4]. Warmer conditions at that time can positively influence pollen production and thereby influence the intensity of the pollen season in the following year [4]–[6], [25]. During the flowering season, weather conditions influence pollen release in tree species that typically flower in late winter and early spring. For example frost may damage the flower buds before pollen release and can therefore reduce the total annual pollen production [1]. These relations differ regionally, as pollen production can be limited by summer temperatures and temperature sum (boreal Finland and Denmark [4]–[6], winter temperature (Jura Mountains [26]), or humidity (Australia [24], Central Europe and Caucasus [25]). Depending on the species, correlations can have opposite signs [25], and reveal autocyclic biological variations in time [2], [23].

To achieve our aims and improve our understanding on climate-pollen relations between different regions and in different types of archives, multiple records of atmospheric and sediment-derived pollen deposition are needed [14]. Also, information on local land use changes and vegetation composition [26], as well as reliable precipitation and temperature data are required. A rare combination of such time series is available in The Netherlands, which presents a unique opportunity to investigate the impact and consistency of climate on annual pollen deposition with exact time control. The correlations and patterns between the different records allow better explanation and independent testing of the annual variability, and thereby help to strengthen long-term allergy forecasts, aid development and understanding of annual-resolved palaeoclimate records, and increase reliability of forensic studies.

## Material and Methods

**Figure 1:**
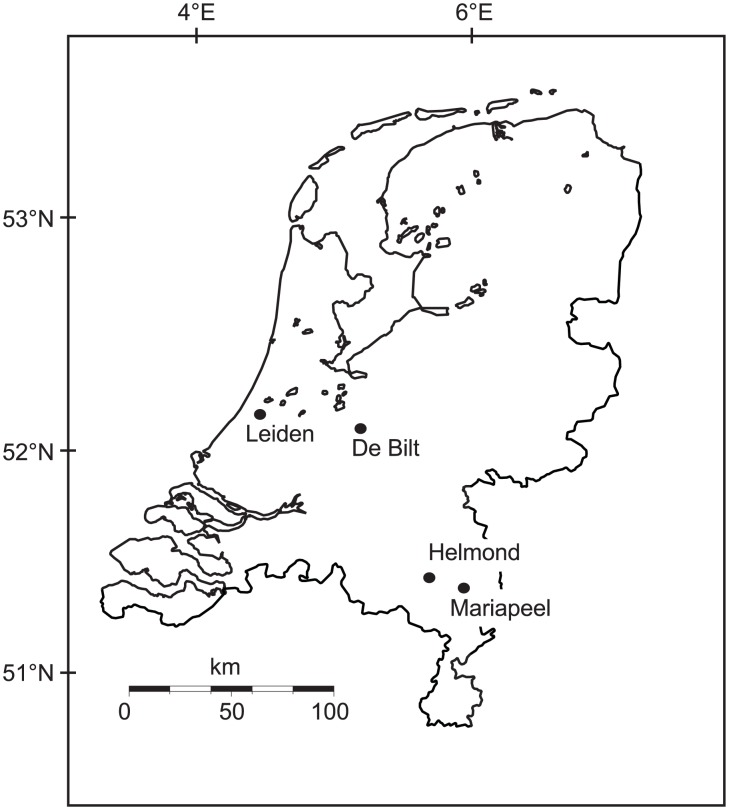
Map of the Netherlands showing the location of the LUMC (Leiden) and Elkerliek (Helmond) pollen traps, De Bilt meteorological station, and the peat deposit from Mariapeel Natural reserve.

At Leiden University Medical Centre (LUMC, 52°23'N; 4°29E) in W Netherlands (Fig. 1, Table S1 in File S1) atmospheric pollen concentrations have been recorded daily since AD 1969 [17], [27], resulting in one of the longest and most continuous pollen trapping stations worldwide. Since AD 1975 a second locality in The Netherlands, the Elkerliek Hospital in Helmond (SE Netherlands, 51°29'N; 5°38E, Fig. 1, Table S2 in File S1), produced daily pollen counts, although counts were not year round until AD 2008. Thus far studies on the pollen-climate relations in the Netherlands have only focused on the influence of climate on the long-term trends and timing of pollen release [17], [27]. The records present a unique opportunity to investigate the impact of seasonal climate variables on annual pollen production as well as assess regional and plant-specific variability in the Netherlands. For example, the degree of consistency between records determines how regionally applicable a pollen forecast is and whether the climatic interpretation of high-resolution palaeovegetation records are robust, and ecosystem dependent. We test effects of seasonal variations in temperature and precipitation on the total annual pollen deposition of anemophilous plants in pollen traps from the Netherlands, and assess regional variations by comparison of both trapping stations and a natural sediment archive.

Comparison with a climate-pollen correlation study from southern Denmark [4] provides a supra-regional view on the most important climate drivers on pollen deposition in NW Europe. Extending the Danish study, we hypothesize that temperature of the growing season in the previous year regionally is the most important factor for annual pollen deposition (used here as the combined result of pollen production and transport processes), and that this factor will be consistent between the different pollen trap records in NW Europe. We expect the natural archive to contain elevated levels of local site-specific vegetation relative to the trapping stations, but the trends and annual-scale patterns to be comparable. Following recommendations by Joosten and De Klerk [28] we indicate pollen taxa in small capitals (e.g., Betula) to distinguish them from taxonomic plant species.

**Figure 2:**
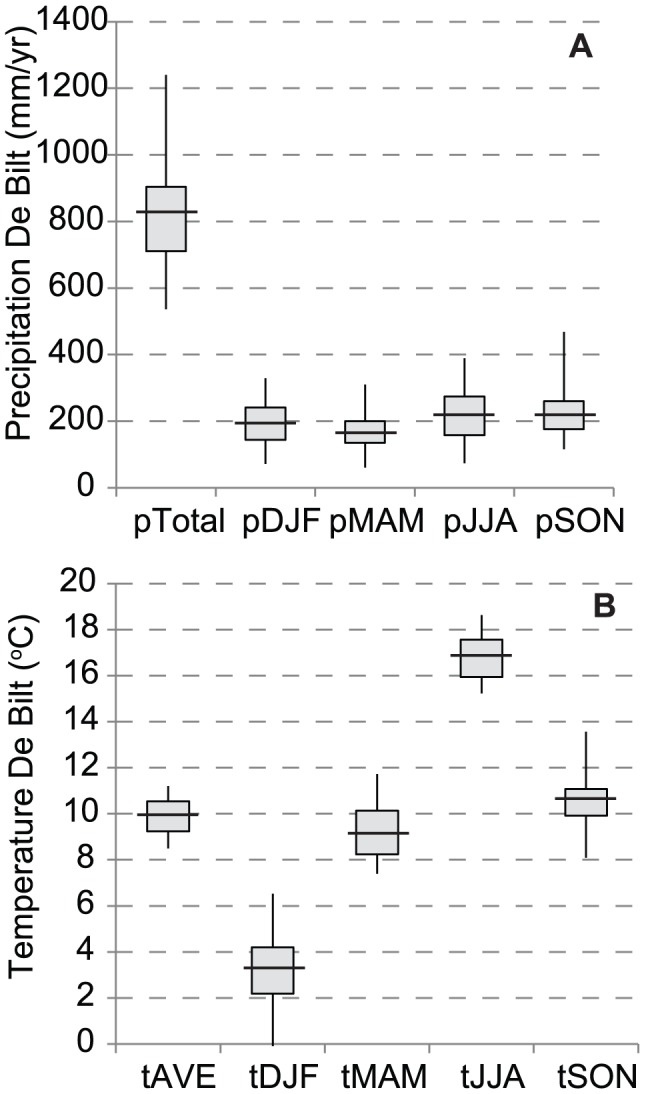
Annual and seasonal variability of the meteorological data from De Bilt. Values are expressed as the upper and lower quartiles, median, and minimum and maximum values of (a) cumulative precipitation and (b) mean temperature for AD 1969–2012.

We use simple linear correlation and multiple linear regression to determine, and statistically model, the influence of monthly and seasonal temperature and precipitation on total annual pollen production. Winter temperature and precipitation in the Netherlands (Fig. 2) are influenced by the NAO, defined by the difference of normalized sea level pressure between Lisbon, Portugal and Reykjavik, Iceland [15]. Therefore, the effect of the winter NAO (December through March) on pollen production is also analysed, as well as testing for cyclic patterns and autocorrelations in the data. Further, a well-dated near-annually resolved pollen record (±1953–1992) from a peat deposit in the vicinity of Helmond [29] provides insight on the reflection of annual-scale variations in pollen production and species-specific offsets in natural archives compared to the trap records. If similar climate-forced variations in annual pollen production can be identified for both the pollen traps and the natural sediment archives, the forcing is regionally relevant and preserves well in natural archives implying that similar high-frequency climate variations can be reconstructed for pre-instrumental periods.

### Source data description

The LUMC trap is located in an urban area with non-natural vegetation in nearby parks, gardens and along streets. In these parks, *Alnus* and *Populus* are the most dominant trees [30]. The Elkerliek area is mostly surrounded by parks and gardens. Pollen was collected with a Hirst-type [31] volumetric, continuous pollen trap [27]. Initially a daily operated type (Castella) was used, while in 1976 (LUMC) and 1980 (Elkerliek) a weekly (Burkard) trap was installed on the roofs ∼200 m from the original location [30]. Daily pollen concentrations are expressed as n m^−3^ day^−1^ in the air, and are corrected for changes in counting and trapping method. Daily pollen counts were summed to determine total annual pollen influx (n m^−3^ year^−1^). The pollen concentration in the air was not measured whole year round at LUMC until 1977 (Table 1), and in Elkerliek until 2008. At LUMC, this caused missing or incomplete data for Corylus counts between 1969 and 1974, and in 1976, and for Alnus counts in 1969, 1973, and 1976 since these species flowered before pollen counts started in those years. For calculation of correlations the missing values on annual pollen influx were replaced by the series mean. Plots of pollen influx for individual taxa against time (Fig. S1) were evaluated for data consistency and missing values, and if needed, corrected based on the original count sheets.

**Table 1:** Availability of LUMC (day of the year, January 1^st^ = day 1).

LUMC pollen data	
Year AD	Measurement period (days)
1969	99–259
1970	75–259
1971	75–259
1972	69–245
1973	71–167
1974	60–167
1975	61–260
1976	63–238
>1977	Year round

A summary pollen diagram from the peat deposit from Mariapeel Nature Reserve nearby Helmond (51^o^ 25' 03.04" N, 5.55' 31.94" E, Fig. 1, Table S3 in File S1) was reported earlier [29], and includes age information and site description. The profile represents secondary peat growth AD ±1953 to 1992 in a small pit after the site was mined in the early 20^th^ century and shows near-annual variability of local and regional vegetation. Age assessment was based on linear interpolation between three local well-documented land-use changes (AD 1961, 1973 and 1980), resulting in a time-scale with an estimated accuracy of ±1 year [29]. Pollen accumulation rates were calculated based on an added spike with a known amount of *Lycopodium clavatum* spores and a fixed sample volume. Initially, counts of 20 pollen types were selected based on anemophily and presence in all three data sources, eight of which are associated with hay fever. LUMC data were used to test correlations with climatic parameters as it is the most complete and consistent, the Elkerliek data are used to assess the regional consistency, and Mariapeel data for comparison between trapping stations and a natural sediment archive. Temperature and precipitation data were obtained from the Royal Netherlands Meteorological Institute (KNMI) at the automatic meteorological station in De Bilt, a central location in the Netherlands (52°06'N; 5°11'E) representative of mean conditions and at intermediate distance between both trapping sites (Figs. 1, 2). The NAO-index data were obtained from the Climate Analysis Section of the National Center for Atmospheric Research, Boulder, USA [32].

### Statistical analysis

Pollen counts were square-root transformed as Q–Q plots and non-parametric Kolmogorov-Smirnov testing showed a non-normal distribution for most pollen types. A principal component analysis (PCA) of the LUMC square-root transformed pollen counts was performed to assess the internal relations of the pollen assemblages using CANOCO software version 4.5 [33]. A two-tailed Spearman rank correlation was performed to assess which pollen types (n = 20), as well as the PCA 1^st^ axis values representative of the pollen assemblage, correlate significantly with climate. All variables, monthly and seasonal temperature (T_month_ and T_season_), precipitation (P_month_ and P_season_), and the NAO index, were tested for both the flowering year and the prior year (subscript _variable-1_). Due to the large number of comparisons, care should be taken for correlations that are only significant at the 95% level as they are likely randomly occurring [4]. Common pollen types with significant correlation to multiple climate variables (see results, Tables 2, 3) were analysed further with a linear regression analysis with time as a predictor, and a multiple regression analysis with climatological variables as predictors.

**Table 2:** Selected pollen types of tree and herb species and their flowering season.

Family	Pollen type	Flowering season
Betulaceae	Alnus*	January, February, March
	Betula*	April, May, June
	Corylus*	January, February, March
Fagaceae	*Quercus*	May, June
Oleaceae	*Fraxinus*	April, May
Plantaginaceae	Plantago*	May, June, July, August, September

Pollen types associated with pollinosis are indicated by *.

**Table 3:** Initial model parameters for multiple regression analysis using backward selection.

Pollen	Predictors
Alnus	January – December Temperature_-1_
	January – April Temperature
Betula	January – December Temperature_-1_
	January – June Temperature
	April Precipitation_-1_
	June Precipitation_-1_
	April Precipitation
Corylus	January – December Temperature_-1_
	January – April Temperature
	Precipitation March_-1_
Fraxinus	January – December Temperature_-1_
	January – June Temperature
Quercus	January – December Temperature_-1_
	January – June Temperature
	Precipitation April
Plantago	January – December Temperature_-1_
	January – September Temperature
PCA 1^st^ axis	January – December Temperature_-1_
	January – September Temperature

Temperature_−1_ stands for temperature in the year before flowering.

An initial model was created for each selected pollen type (n = 6), including monthly temperature values of the year before flowering (i.e. lag −1), and monthly temperature during the current year until one month after the end of the flowering season (following ref. [34]) (Table 3). Precipitation variables were only included when the Spearman rank correlation showed a significant correlation to pollen influx. To prevent statistical over fitting, a stepwise backward multiple regression analysis was performed based on the initial models to reduce the number of redundant parameters. While stability of weather patterns can cause several consecutive months to correlate with pollen production, only a single or few months are relevant for pollen production [25]. Prediction skill of the resulting model was tested by random division of the dataset in a calibration and an independent test set. Predicted values were plotted against observed values, whereby R^2^ and associated P-values <0.05 were considered to be statistically significant models. Cyclicity patterns and autocorrelations were evaluated through multitaper spectral analysis method incorporated in PAST software version 2.17 [35], which produces an F-value statistic for significance testing.

## Results

### General trends

**Figure 3:**
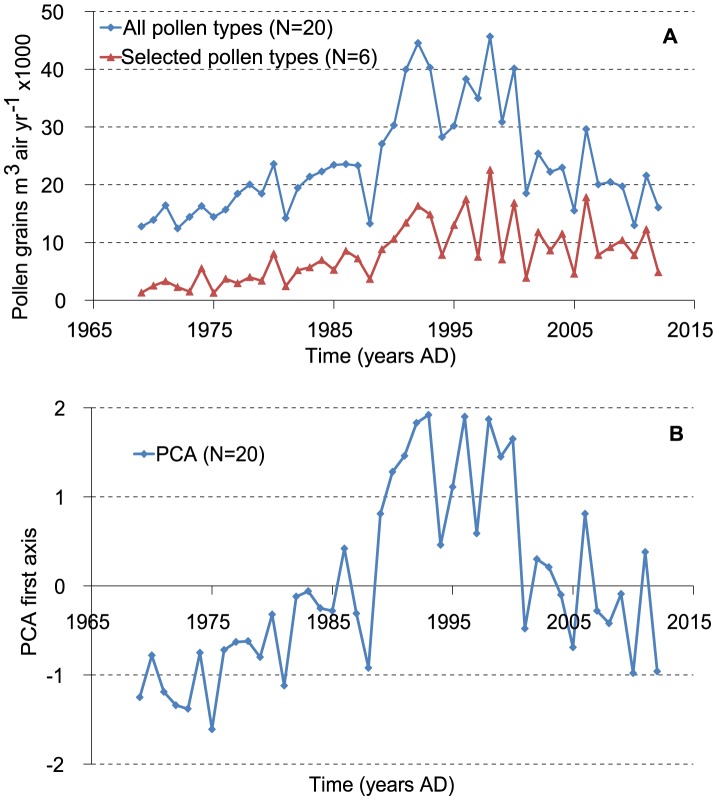
Total annual pollen influx values 1969–2012. (a) Total LUMC pollen influx for all pollen types (n = 20) and for selected pollen types (n = 6), and (b) sample values of the 1^st^ PCA axis based on square-root transformed pollen count data.

**Figure 4:**
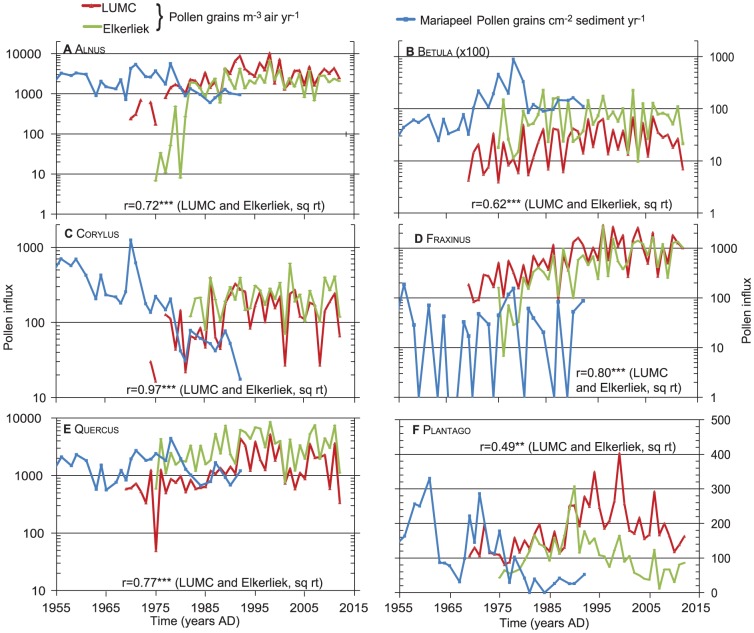
Annual pollen influx values 1969–2012 for LUMC, Elkerliek (n m^−3^ air yr^−1^) and Mariapeel (n cm^−2^ sediment yr^−1^). Values are shown for (a) Alnus, (b) Betula, (c) Corylus, (d) Fraxinus, (e) Quercus, and (f) Plantago. Note that a–e are on a logarithmic y-axis.

The total pollen influx of all pollen types (n = 20) at LUMC ranges from 12.5×10^3^ to 45.7×10^3^ m^−3^ year^−1^ with an average pollen influx of 23.5×10^3^ m^−3^ year^−1^(Fig. 3a). Long-term influx of Alnus, Carpinus, Corylus, Quercus, Juglans and Fraxinus pollen increases from about AD 1990, while Tilia is more dominant before AD 1990 (see Fig. 3b and Fig. S1). The herb pollen types Poaceae, Artemisia, Chenopodiaceae, Plantago and Urticaceae show a phase of maximal influx between approximately AD 1990 and 2000. For 20 pollen types tested, only 6 show no significant correlation to any climate parameter (Tables S4–8 in File S2). Correlations are clearly stronger and more frequent for the temperature variables. In contrast to precipitation, within-season variability is much smaller than between-season variability for temperature (Fig. 2), but the within-season effectively controls the length of the seasons. Six common pollen types with multiple significant correlation coefficients and consistent records at both pollen trap stations, Alnus, Betula, Corylus, Fraxinus, Quercus and Plantago (Table 2), were selected for further regression analysis (Tables 4–6, Fig. 4). Year-to-year deposition of the six pollen types at LUMC and Elkerliek vary consistently with highly significant correlations of the square root-transformed data (Fig. 4). A linear regression of the total influx shows significant long-term increase with time for all six selected pollen types except Corylus for the LUMC site, while only Fraxinus increases significantly at Elkerliek (Table 4).

**Table 4:** Linear regression of total annual of pollen influx with time (year) as predictor.

	*LUMC, Leiden*				*Elkerliek, Helmond*			
Pollen	*M*	*S.E.*	*r^2^*	*β*	*M*	*S.E.*	*r^2^*	*β*
Alnus	2926	354	.278	.527***	2241	234	.037	.191
Betula	2643	287	.144	.379*	7880	963	.010	.100
Corylus	147	16	.072	.268	233	22	.010	.102
Fraxinus	841	110	.387	.622***	653	96	.456	.675**
Quercus	1469	183	.225	.474***	3558	361	.091	.302
Plantago	180	11	.201	.448**	106	10	.095	−.308

Significance levels are indicated: * P<0.05; ** P<0.01; ***P<0.001. S.E =  standard error, M =  mean, β =  slope.

**Table 5:** Correlation coefficients between annual total pollen influx per pollen type, temperature and NAO index as shown by a Spearman Rank Correlation.

			Spearman rank correlation (r_s_), 2-tailed						
Climate variable			Alnus	Betula	Fraxinus	Corylus	Quercus	Plantago	PCA 1^st^ axis
Monthly temperature	Previous year	T_jan-1_	.240	.134	.129	−.024	.128	.248	.229
		T_feb-1_	.195	−.006	.324*	.158	.046	.318*	.167
		T_mar-1_	.530***	.197	.512***	.309	.360*	.487***	.459**
		T_apr-1_	.360*	−.014	.513***	.048	.173	.360*	.145
		T_may-1_	.170	.308*	.193	.224	.144	.444**	.297
		T_jun-1_	.124	.317*	.037	−.037	.110	.019	.090
		T_jul-1_	.318*	.564***	.371*	.562***	.440**	.192	.442**
		T_aug-1_	.363*	.237	.290	.348*	.135	.048	**.**260
		T_sept-1_	.054	.072	.064	.079	.124	.175	.062
		T_oct-1_	.016	.093	−.010	.152	−.046	−.002	.031
		T_nov-1_	.138	.200	.081	−.032	.005	−.036	.035
		T_dec-1_	−.044	.109	.030	−.062	.070	.318*	.098
	Flowering year	T_jan_	−.040	.078	.130	−.090	.201	.209	.084
		T_feb_	.117	.320*	.167	.143	.494***	.278	.245
		T_mar_	.329*	−.022	.385**	.277	.147	.500***	.330*
		T_apr_	.507***	.463**	.393**	.325*	.576***	.303*	.430**
		T_may_		.093	.224		.510***	.440**	.314*
		T_jun_		.171	.241		.272	.244	.206
		T_jul_						.318*	.143
		T_aug_						.179	.152
		T_sept_						.131	.150
Seasonal temperature	Previous year	T_winter-1_	.225	.136	.227	.083	.169	.293	.260
		T_spring-1_	.506***	.254	.597***	.295	.290	.592***	.422**
		T_summer-1_	.365*	.534**	.306*	.399*	.362*	.113	.372*
		T_autumn-1_	.146	.175	.135	.150	.031	.096	.080
	Flowering year	T_winter_	−.004	.219	.153	−.027	.328*	.355*	.176
		T_spring_	.534***	.200	.452**	.350*	.572***	.573***	.476***
		T_summer_		.114	.314*		.139	.349*	.223
NAO	Previous year	NAO_-1_	.178	.085	.054	−.117	−.041	.199	.168
		NAO_DJFM-1_	.272	.242	.202	−.029	.184	.448**	.376*
	Flowering year	NAO	.197	.251	.296	.279	.373*	.466**	.401**
		NAO_DJFM_	.100	.160	.114	.019	.209	.248	.290

Significance levels are indicated: * P<0.05; ** P<0.01; ***P<0.001.

**Table 6:** Correlation coefficients between annual total pollen influx per pollen type and precipitation as shown by a Spearman Rank Correlation.

			Spearman rank correlation (r_s_), 2-tailed						
Climate variable			Alnus	Betula	Fraxinus	Corylus	Quercus	Plantago	PCA 1^st^ axis
Monthly Precipitation	Previous year	P_jan-1_	.161	−.051	.030	−.153	−.124	−.043	.017
		P_feb-1_	.163	.095	.203	.190	.044	.144	.074
		P_mar-1_	−.037	.238	−.074	−.042	.241	.110	.147
		P_apr-1_	−.246	.407**	−.042	.288	.186	.044	.110
		P_may-1_	−.089	.138	.029	−.172	.114	−.048	−.083
		P_jun-1_	−.054	−.326*	.130	−.041	−.119	.173	−.098
		P_jul-1_	−.038	−.283	.095	−.205	−.074	.030	−.208
		P_aug-1_	.002	.064	−.021	.226	.181	.079	.016
		P_sept-1_	.211	.090	.215	−.037	.218	.065	.185
		P_oct-1_	.006	−.045	−.075	−.241	.056	.024	.070
		P_nov-1_	−.198	−.090	−.102	.111	.048	−.048	−.064
		P_dec-1_	.019	.196	.253	−.138	−.012	.176	.114
	Flowering year	P_jan_	−.241	.066	.006	−.216	.058	−.053	−.068
		P_feb_	−.117	.186	.057	.053	.144	.029	.021
		P_mar_	−.085	−.052	−.139	−.340*	.195	.183	−.005
		P_apr_	−.080	−.308*	−.148	−.143	−.333*	.175	−.072
		P_may_		−.154	−.138		−.129	−.030	−.160
		P_jun_		.037	−.120		.123	−.008	.104
		P_jul_						−.136	−.114
		P_aug_						.030	−.029
		P_sept_						.229	.168
Seasonal Precipitation	Previous year	P_winter-1_	.189	−.014	.083	−.076	−.033	.049	.061
		P_spring__-1_	−.204	.375*	−.054	−.004	.312*	.060	.086
		P_summer-1_	−.025	−.208	.094	.034	.083	.208	−.091
		P_autumn-1_	.066	−.014	.032	−.116	.195	.037	.130
	Flowering year	P_winter_	−.188	.194	.143	−.166	.068	.069	.012
		P_spring_	−.142	−.303*	−.259	−.334*	−.126	.162	−.139
		P_summer_		.068	.052		.271	.003	.001

Significance levels are indicated: * P<0.05; ** P<0.01; ***P<0.001.

### Correlation to climate

Positive significant relations were found between the NAO index and the annual pollen influx of Quercus and Plantago, as well as the first axis of the PCA analysis, which represents mean composition of the pollen assemblage. Positive significant influence of mean growing season temperature, of the year prior to the flowering season (T_summer-1_ and /or T_spring-1_) is evident in all six pollen types (Table 5), where Alnus, Fraxinus and Plantago influx show particularly strong relations with T_spring-1_, while T_summer-1_ is important for Betula and, to a lesser degree, Corylus and Quercus. During the year of flowering, T_spring_ is dominant except in Betula. Based on the monthly correlations, April and March are the most important months in T_spring_ and T_spring-1_, while T_jul-1_ is the dominant summer month. The only herbaceous type, Plantago is also significantly influenced by T_may_ and T_may-1_. Correlations show much less influence of precipitation on total pollen influx values (Table 6). Only Betula influx is positively influenced by P_spring-1_ (mainly P_apr-1_), while in both Betula and Corylus P_spring_ (for Betula: P_apr_ and Corylus: P_mar_) has a negative influence, although not highly significant and possibly a result of random correlations.

### Predictive regression model

**Figure 5:**
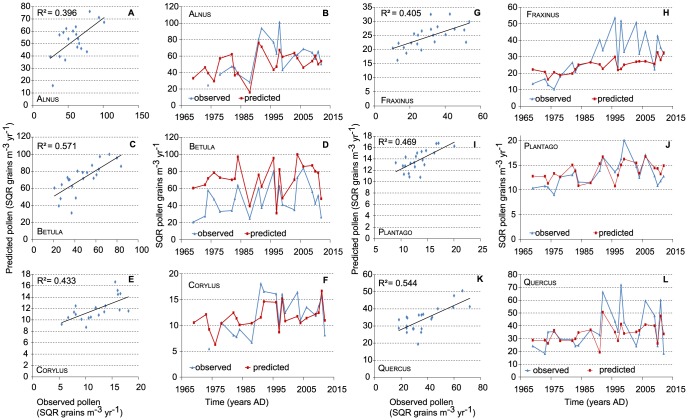
Observed versus climate predicted LUMC pollen influx based on the split data sets. Values are shown for (a,b) Alnus, (c,d) Betula, (e,f) Corylus, (g,h) Fraxinus, (i,j) Quercus, and (k,l) Plantago.

**Figure 6:**
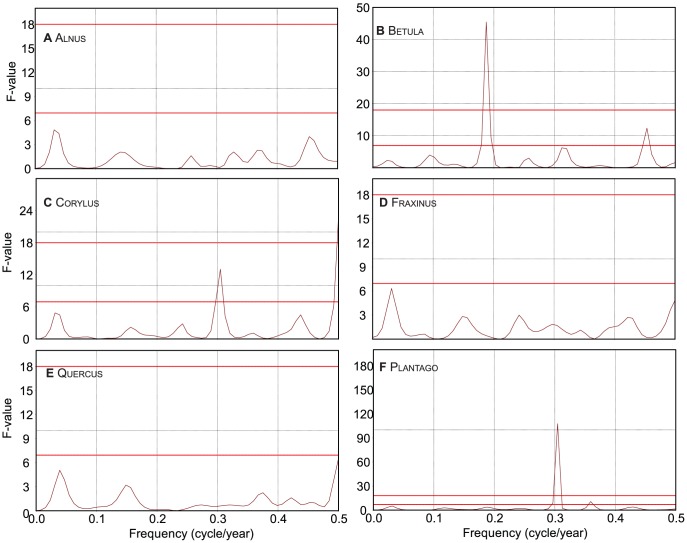
Spectral analysis output with 95% and 99% significance levels (upper and lower red line, respectively) of the 1969-2012 annual pollen influx time series in the LUMC trap. (a) Alnus, (b) Betula, (c) Corylus, (d) Fraxinus, (e) Quercus, and (f) Plantago.

The effect of a combination of meteorological variables on pollen influx, assessed through multiple regression analysis with backward selection, is shown in Table 7 for the LUMC data. Calibration and prediction skill of the resulting optimal model of pollen influx are shown in Fig. 5. The optimal regression models show a good performance based on their reported R^2^. In Betula and Fraxinus some redundant parameters were discarded after the data splitting step, creating a model with slightly lower performance but less parameters, thereby reducing the complexity and statistical overfitting. Growing season temperature of the previous year again shows to be the most important factor, especially T_mar-1_. In addition, winter temperatures prior to flowering show to be significant as well (T_nov-1_, T_dec-1_, T_jan_, T_feb_) and mostly have a negative loading (Table 7). Depending on the genus, T_mar_ has a negative (Betula) or positive (Alnus, Plantago) loading in the model. Plotted against time (Fig. 5), the trends and phase relations between the observed and predicted influx values correspond well, especially for Betula and Plantago, although the variability in the predicted values is usually slightly lower than the observations. The multitaper spectral analysis showed significant cyclic patterns for Betula (Fig. 6b) at a frequency of 0.19 (5.3 year) and 0.45 (2.2 year). Also Corylus (3.3 and 2 year, Fig. 6c) and Plantago (3.2 and 2.6 year, Fig. 6f) show significant variability at short periodicities.

**Table 7:** Statistical parameter values of the regression model with backward selection method and the optimal regression model after data splitting.

Pollen type	Regression model after backward selection			Optimal regression model after data splitting analysis		
	*R^2^*	Variable	β	*R^2^*	Variable	β
Alnus	.538***	T_March-1_	.549***	.538***	T_March-1_	.549***
		T_August-1_	.303**		T_August-1_	.303**
		T_January_	−.265*		T_January_	−.265*
		T_March_	.230		T_March_	.230
Betula	.677***	T_January-1_	−.258*	.565***	T_March-1_	.286*
		T_March-1_	.432***		T_May-1_	.333**
		T_May-1_	.411***		T_July-1_	.486***
		T_July-1_	.410**		T_August-1_	.195
		T_August-1_	.263*		T_November-1_	−.248
		T_October-1_	.209		T_December-1_	.261*
		T_November-1_	−.327*		T_March_	−.358**
		T_December-1_	.298*		P_June-1_	−.357**
		T_February_	.225			
		T_March_	−.513***			
		P_June-1_	−.363**			
Corylus	.616***	T_January-1_	−.377**	.616***	T_January-1_	−.377**
		T_March-1_	.330**		T_March-1_	.330**
		T_May-1_	.276*		T_May-1_	.276*
		T_July-1_	.437***		T_July-1_	.437***
		T_August-1_	.219		T_August-1_	.219
		T_October-1_	.261*		T_October-1_	.261*
		T_November-1_	−.364**		T_November-1_	−.364**
		P_march_	−.224*		P_march_	−.244*
Fraxinus	.629***	T_January-1_	−.298*	.386***	T_February-1_	.325*
		T_February-1_	.438**		T_April-1_	.376**
		T_March-1_	.392**		T_July-1_	.298*
		T_April-1_	.414***			
		T_July-1_	.475***			
		T_November-1_	−.232			
		T_January_	−.340**			
Quercus	.703***	T_March-1_	.296**	.523***	T_July-1_	.493***
		T_July-1_	.498***		T_May_	.544***
		T_November-1_	−.219*			
		T_January_	−.242*			
		T_April_	.242*			
		T_May_	.514***			
Plantago	.579***	T_March-1_	.254*	.463***	T_March-1_	.277*
		T_May-1_	.211		T_May-1_	.196
		T_November-1_	−.212		T_March_	.298*
		T_March_	.230		T_July_	.276*
		T_May_	.264*			
		T_July_	.289*			

Significance levels are indicated: * P<0.05; ** P<0.01; *** P<0.001.

## Discussion

### General observations

The primary aim of this study was to investigate the possible effects of temperature and precipitation variables on the annual pollen production of wind-pollinated plants in the Netherlands. Earlier evaluations showed no significant long-term increase for Betula and Quercus [30], however, ten years of extra data has revised this conclusion (Table 4). The long-term trend observed in Fig. 3b can in part be attributed to construction work at the LUMC site from 1985 until the mid-1990s, which provided favourable conditions for early successional herb species on cleared land. Increased planting in recent years of non-native *Alnus spaethi*, which is well-adapted to urban environments and has an early flowering season, probably adds to the long-term increase as well. Explanation of the increased pollen influx observed in most taxa (see Fig. S1) in terms of long-term temperature change is tempting, considering the sensitivity to annual-scale temperature variations shown in Table 5, but other factors such as long-term increase of CO_2_ [7], [36], potentially increased production due to long-term atmospheric nutrient deposition [37], [38], as well as non-documented changes in composition and management of the urban vegetation cannot be discarded.

The results of this study showed that the annual pollen influx of Alnus, Betula, Corylus, Fraxinus and Quercus, was positively influenced by summer temperature in the year before flowering, which coincides with the production of flowering buds for most tree species [1], [4] and confirms our hypothesis. The reported relation between annual influx of Alnus, Betula, Fraxinus, Quercus and Plantago and temperature during the flowering season is likely more related to the final stages of pollen ripening and deposition speed. In that light, it is surprising that precipitation has little effect on most taxa (Table 6), in contrast to the conclusions for central Europe [25]. Only for Betula the effect of additional precipitation is beneficial for the following year, while spring rain during pollen release reduces atmospheric pollen content [4], although it is unclear why this is only for Betula and Corylus in our data. The Netherlands rarely has significant water shortage (Fig. 2), and water stress is therefore an unlikely limiting factor for pollen production for most taxa. Comparison of the correlation coefficients in Tables 5 and 6 with results from Southern Denmark [4] show that particularly pollen deposition of Betula, Fraxinus and Quercus are driven by largely the same climatic variables, while Corylus agrees in sensitivity to T_apr_ and T_apr-1_, but not T_jan/feb_ (Denmark) and T_jul-1_ (LUMC). Contrary to the LUMC data, Alnus in Denmark shows surprisingly little correlation with climatic variables. Clearly, these results highlight the need for region-specific correlation models for pollen-climate relationships, but do identify climate factors of regional relevance. Specifically for the medical treatment of pollinosis, the linear models (Table 7 and Fig. 5) are a first step in the development of a seasonal predictive model for pollen forecasting. A subsequent step should involve combining the total season pollen load with knowledge on the timing of pollen release, which is climate dependent [17], [39]. For forensic palynology, the most important aspect of the study is the fact that the pollentrap record shows variability that is consistent across large distances (Fig.4), which corroborates a study from the Jura Mountains in which pollen composition from several high-resolution peat records were compared [26]. Annual variations in pollen deposition and composition are largely climate driven and, hence, it is meaningful to compare samples from a crime scene to a pollen trap record to estimate ages or relative timing (season).

#### Alnus

In *Alnus*, catkins are initiated in early July and meteorological conditions before this period can be expected to influence their formation [40]. In this study, the positive influence of T_mar-1_ suggests that warmer March temperatures stimulate catkin formation in *Alnus*. Catkin formation is followed by a dormancy period during autumn and winter, which protects them from frost damage, until temperature increases at the start of pollen season [40], [41]. For the United Kingdom, it has been suggested that bud dormancy starts in August and lasts until February [40]. However, the results of this study indicated that T_aug-1_ affects Alnus production, suggesting that catkin formation continues in August and bud dormancy starts in the period after August in the Netherlands. A study on the Iberian Peninsula suggested that the chilling period for *Alnus* starts in November and continues until January [41].

In the Netherlands, anthesis (opening of flower buds) and pollination takes place from January until March [34]. The relation of T_mar_ with Alnus influx found here suggests that warmer temperatures during the flowering season stimulate pollen release or lengthens the flowering season. In the Netherlands, it has been demonstrated that T_jan_ affects the start of the Alnus pollen season [39], although the Danish results showed no clear relation with temperature [4]. Despite a reported biannual cyclicity for Alnus [23], [41], our study (Fig. 6a) and results from Denmark and Spain showed no significant cyclic patterns [4], [41], although some autocorrelation is present with a 2 year time lag (Fig. S2), pointing to a limited role of autocyclic processes in *Alnus* flowering intensity.

#### Betula

In the Netherlands *Betula* species occur: *Betula pendula* and *B. pubescens*. *B. pubescens* is closely related to *B. papyrifera* [18] and the phenology of the latter has been thoroughly investigated [42]–[44]. The phenology of *B. papyrifera* is also characteristic to *B. pendula* and *B. pubescens* [18] and can thus be used as a general description of flower formation of *Betula* trees in the Netherlands. The initiation of male catkins starts just before leaves develop in the year before flowering. They become visible in June and July and develop until August after which dormancy begins [18], [43], [44]. In the Netherlands, leaf unfolding generally begins in April [45]. It can thus be expected that the formation of male catkins begins in April and that the complete catkin development likely continues until August.

The here reported relation of Betula influx with T_mar-1_, T_may-1 and_ T_jul-1_ coincides with the main period of catkin formation. Studies in Denmark show correlations to T_may-1_, T_jun-1,_ T_jul-1_ [4], [48], and in northern Finland to T_jun-1_ [6], suggesting an earlier start of catkin formation more to the south. The sensitivity of Betula to precipitation (P_apr/jun-1_) is identical to that in Denmark [4], and similar to central Europe [25], and might be related to its preference for relatively wet habitats, particularly in *B. pubescens*. The 5-year cyclicity found in Betula only, possibly relates to precipitation as well (Fig. 5b). Here, we hypothesize that *Betula* invests in reproduction strategies (i.e. pollen production) during dry conditions, while in wetter conditions these trees invest in vegetative growth.

Including the temperature values of the October - March winter dormancy period in *Betula* [18] improves the predictive model (Table 7). It can be hypothesized that warmer temperatures in November may disturb this winter dormancy but as the sign of the correlation differs between November and December this needs independent confirmation. The negative relation with T_mar_ is remarkable as *Betula* flowering season is between April and June [34]. A possible explanation might be that although warmer temperatures in early spring can advance the *Betula* pollen season [17], the increased chance of frost damage results in lower annual pollen influx.

Beside the strong 5-year cyclicity, a 2.4 year cycle in Betula influx confirms earlier finds of bi-annual variability [18] and a three-year cycle [21]. As high pollen production likely results in a high energy-intensive fruit production this might inhibit the development of reproductive structures for the flowering season in the next year [30]. Ranta et al. [2] indeed conclude that “masting of birch species is regulated by weather factors together with the system of resource allocation among years”. High inflorescence numbers might result in smaller and fewer leaves, lowering overall photosynthetic capacity of the tree including development of new flower buds. As a consequence, few inflorescences in the following year will relocate more energy for the development of leaves, and in turn stimulates the development of flower buds [18]. Pollen production is indeed correlated to catkin formation, and year-to year changes have previously been shown to be similar across large distances (up to 500 km) [49].

As temperature and precipitation influence photosynthetic capacity, climatic conditions are likely to exert control over this autocyclic pattern, which might explain variations between 2- and 3-year cycle lengths.

#### Corylus

As in Betula, Corylus pollen influx broadly depends on growing season temperature of the year before and shows an earlier start compared to Denmark [4]. *Corylus* flowers early in the year, from January until March [34], and unlike observations in Denmark [4], Corylus influx has no (negative) relation with respect to winter temperatures, which confirms their cold adaptation and relative insensitivity to temperature in that period in this region. The negative effect of P_mar_ on annual pollen influx is probably the consequence of rain washing the pollen out of the atmosphere during the pollination period. The 2.3 year cyclicity in the signal is of the same character as in Betula (Fig. 6c), and has also been observed in the United Kingdom [22]. Although no specific studies confirm this, we infer a similar mechanism as that described for *Betula* as both are Betulaceae.

#### Fraxinus

The factors T_feb-1_, T_apr-1_ and T_jul-1_ best predict the annual Fraxinus influx suggesting a particularly long period of flower bud formation and sensitivity. Highly similar results from Denmark confirm this [4], while a study from Galicia (northwest Spain) involves also precipitation as an important factor for *Fraxinus* flowering [8]. The predictive model does not explain the majority of the variability (39%), suggesting edaphic factors and other internal biological processes play a significant role.

#### Quercus

In the temperate climate zone, *Quercus* forms flower buds during the summer of the previous year and enters a dormancy period during autumn and winter [46].The sensitivity to T_jul-1_ at LUMC and T_aug-1_ in Denmark [4] suggest a short critical period for bud formation. In cork-oak (*Q. suber*), reports of steep temperature drop during initial stages of microsporogenesis resulted in catkin mortality and much lower pollen quantities [16].

#### Plantago

In perennial herbaceous plants, warm springs and summers in previous years are important for plant growth and flower development, as shown by the influence of T_mar-1_ and T_may-1_in our analysis, whereas summer droughts may kill plants and thereby affect the pollen production [23]. Warmth during early spring and summer stimulates plant development and flower differentiation [23] and continued pollen generation typical of herbs [47], which can explain the positive impact of T_mar_ and T_Jul_ on the annual influx of Plantago. Earlier studies of *P. lanceolata* from Poland did not reveal climate/pollen relationships, likely limited by the length of the record (10 years) [25]. The results of this study also show a positive correlation between the NAO index and the total annual Plantago influx. The multi-taper analysis of the annual Plantago influx reveals a particularly strong 3.2- year cycle (Fig. 6f), which, in combination with a positive correlation with the NAO index, points to a multi-annual climatic influence.

### Palaeoecological interpretation

Our aim to compare trends in pollen trap data from LUMC and Elkerliek with those in the palaeoecological record are complicated by human management of the Mariapeel area and, despite the accurate depth-age model, slight age uncertainties that preclude a year-to-year correlation between the trapping and sub-fossil data. The two pollen trap sites agree surprisingly well in terms of phase relation and absolute numbers of pollen (Fig. 3). The Elkerliek site has generally higher amounts of Betula and Quercus, which is in agreement with the greater amount of tree cover, sandy soils and vicinity to natural reserves of that site. LUMC has a greater amount of Plantago, in agreement with a more urban setting. The offset in Alnus of the early part of the record in Elkerliek is related to incomplete counts in the first years. The consistency and significant correlations between both trap data (Fig. 4) shows that the stations produce data that are representative of a broad region. This suggests that the correlation with climate quantified at LUMC is a regionally relevant ecological factor that is responsible for the majority of the observed annual-scale pollen influx variability. This observation also aides comparison of pollen assemblages with the trapping stations in forensic studies (e.g. for the purpose of dating a crime scene).

The Mariapeel site presently is a semi-open *Betula* forest with secondary *Sphagnum* growth in pits formerly mined for peat. The pollen accumulation rates from the sub-fossil peat deposit show a large decrease in all taxa around AD 1980 when the area was declared nature reserve and much of the local standing *Betula* tree vegetation has been cut and water levels were raised to stimulate peat regrowth. Visual inspection of the pre-1980 data (Fig. 3) from Mariapeel show variations of the same order (frequency and amplitude) as the variability present in the pollen trap records. Pollen from the more regionally occurring trees, such as *Quercus* and *Corylus*, show comparable trends and short-term changes as the trap data, but the local changes and short overlap period between unimpacted local vegetation and the traps preclude detailed correlation. The comparison does demonstrate that natural (peat) archives record and preserve high-order variations, with similar amplitude and frequencies as in the pollen trap data. Given the preserved high frequency variations and the identified climatic drivers (Tables 5 and 6) of annual pollen deposition, natural archives can provide significant insights in past climatic variations at near annual scales (see e.g. [13]). Hence, our analysis provides a regional interpretation framework for climatic interpretations of high-frequency changes in pollen records in NW Europe.

## Conclusion

This study showed that climate is an important factor in annual pollen production in the Netherlands and that annual pollen influx shows highly similar variability across a broad geographical area, which is driven by largely the same variables. Summer temperatures in the year before flowering, as well as temperature during the flowering season, are the primary climate variables that determine the annual pollen influx of wind-pollinated plants, while the effect of precipitation is minimal, except for Betula. Summer temperature influences the formation of reproductive structures, while temperature during the flowering season is thought to influence pollen release. The importance of long observational records is evident as shorter series often contain too much scatter to determine pollen-climate relations. Our results provide a first developmental step toward a region-specific predictive model for seasonal pollen forecasting for hay fever patients and forensic studies, and on short timescales (years to decades) predicts the likely impact of changing temperatures on annual pollen production due to global change.

The similar-scale high frequency variations observed in the peat record compared to the pollen traps suggest that, although influenced by local edaphic factors, natural archives can provide a proxy for quantitative reconstruction at high (e.g. annual) temporal resolution. However, the observed relation between climate and pollen production found for the pollen data from Leiden cannot be directly tested on the samples of from the Mariapeel, due to local landscape management changes and small differences in age.

## Supporting Information

Figure S1 **Total annual pollen accumulation rates of all recorded taxa in the pollen traps from LUMC, Leiden, and climate variables from De Bilt, The Netherlands from 1969 to 2012.**
(EPS)

Figure S2 **Autocorrelation of LUMC pollen accumulation rates for 0 to 20 years lags. Curved lines represent 95% significance level.**
(EPS)

File S1 **This file contains Table S1–Table S3.** Table S1. Pollen count data for LUMC pollen traps. Table S2. Pollen count data for Elkerliek pollen traps. Table S3. The peat core from Mariapeel Natural Reserve, The Netherlands. For locations see main text and Fig. 1.
(XLSX)

File S2 **Spearman rank correlation coefficients between annual pollen influx and climate variables of the pollen types not shown in** **Tables 5** **and** **6** **of the main text (Tables S4–S8).** Table S4. North Atlantic Oscillation Index (annual and winter). Table S5. Temperature in the flowering year. Table S6. Temperature in the year before flowering. Table S7. Precipitation in the flowering year. Table S8. Precipitation in the year before flowering.
(XLS)
